# The Histone Deacetylase 9 Stroke-Risk Variant Promotes Apoptosis and Inflammation in a Human iPSC-Derived Smooth Muscle Cells Model

**DOI:** 10.3389/fcvm.2022.849664

**Published:** 2022-03-30

**Authors:** Alessandra Granata, Ioannis Kasioulis, Felipe Serrano, James D. Cooper, Matthew Traylor, Sanjay Sinha, Hugh S. Markus

**Affiliations:** ^1^Stroke Research Group, Department of Clinical Neurosciences, University of Cambridge, Cambridge, United Kingdom; ^2^Anne McLaren Laboratory, Wellcome Trust-MRC Cambridge Stem Cell Institute, University of Cambridge, Cambridge, United Kingdom; ^3^UK Dementia Research Institute, University of Edinburgh, Edinburgh, United Kingdom; ^4^Queen Mary University of London, London, United Kingdom; ^5^Department of Medicine, Wellcome - MRC Cambridge Stem Cell Institute, University of Cambridge, Cambridge, United Kingdom

**Keywords:** HDAC9, human induced pluripotent stem (hiPS) cells, ischemic stroke, smooth muscle cells, risk variant

## Abstract

A common variant in the Histone Deacetylase 9 (*HDAC9*) gene is the strongest genetic risk for large-vessel stroke, and HDAC9 offers a novel target for therapeutic modulation. However, the mechanisms linking the *HDAC9* variant with increased stroke risk is still unclear due to the lack of relevant models to study the underlying molecular mechanisms. We generated vascular smooth muscle cells using human induced pluripotent stem cells with the *HDAC9* stroke risk variant to assess HDAC9-mediated phenotypic changes in a relevant cells model and test the efficacy of HDAC inhibitors for potential therapeutic strategies. Our human induced pluripotent stem cells derived vascular smooth muscle cells show enhanced *HDAC9* expression and allow us to assess HDAC9-mediated effects on promoting smooth muscle cell dysfunction, including proliferation, migration, apoptosis and response to inflammation. These phenotypes could be reverted by treatment with HDAC inhibitors, including sodium valproate and small molecules inhibitors. By demonstrating the relevance of the model and the efficacy of HDAC inhibitors, our model provides a robust phenotypic screening platform, which could be applied to other stroke-associated genetic variants.

## Introduction

Ischemic stroke is a complex disorder caused by a combination of multiple genetic and environmental factors.

Recent GWAS studies have identified multiple loci associated with stroke risk (1), but none of these discoveries have yet led into therapeutic advances. A major obstacle has been understanding the consequences of these associations, and developing disease relevant models in which therapeutic interventions can be screened.

Large-artery stroke, usually caused by intra- and extracranial large-artery atherosclerosis, accounts for a quarter of all ischemic strokes (2). Despite a number of effective secondary prevention therapies such as statins, and anti-platelet agents, many recurrent strokes still occur, and it is the stroke subtype associated with the highest risk of stroke recurrence (3). Thus, new and relevant models are required to gain insights into pathogenesis and to develop additional preventative therapies.

Here, we developed a human induced pluripotent stem cell (hiPSC) model for the GWAS locus in the Histone Deacetylase 9 (*HDAC9*) gene region, which has been most strongly associated with ischemic stroke, to assess phenotypic and molecular changes and test inhibitors for potential therapeutic interventions (4). A rs2107595 single-nucleotide polymorphism (SNP) outside the 3′ end of *HDAC9* gene has been identified as the likely causal variant, and the association between this variant and large artery stroke has been consistently replicated in other stroke patient cohorts (1, 5).

The rs2107595 SNP has also been associated with asymptomatic carotid plaque (6), coronary artery disease (7), and vascular calcification (8). Increased *HDAC9* mRNA levels have been identified in human carotid atherosclerotic plaques, and knockout of *HDAC9* in a mouse model for atherosclerosis, the apolipoprotein E-deficient (ApoE-/-) mouse, was associated with reduced aortic atherosclerosis (9). All these lines of evidence support a pro-atherogenic role for HDAC9.

Histone Deacetylase 9 is a member of the class IIa HDAC subtype within the large family of HDACs (10). These enzymes play a crucial role in many physiological and pathological processes, by interacting with tissue-specific transcription factors to repress/de-repress target genes in specific cell types. HDAC9 is expressed in a variety of cell types linked to cardiovascular diseases, including cardiac cells, T lymphocytes, adipose tissue and smooth muscle cells (SMC) (6, 11). HDAC9 also appears to play a significant role in the immune system (12).

Histone Deacetylase 9 inhibition therefore represents a potential drug target to reduce stroke. In support of this the anti-epileptic sodium valproate, which has pan-HDAC inhibitory properties, reduced atherosclerosis in a rabbit model, and epidemiological studies have reported that sodium valproate is associated with reduced stroke risk compared with other anti-epileptic drugs (13). However, it is unclear which cells within the plaque are mostly affected by the HDAC9 risk variant and how cells phenotypic changes linked to increased HDAC9 could contribute to the pathology; thus, further studies are required to assess the effects of the vascular risk attributed to HDAC9 in a new biologically relevant system, which importantly could provide an easy readout for phenotypic drug screening.

To address this, we developed a relevant vascular smooth muscle cells (SMC) model by using hiPSC harboring the *HDAC9* stroke-associated risk variant to assess HDAC9-mediated phenotypic changes and screen the efficacy of HDAC inhibitors.

## Results

### Histone Deacetylase 9 Was Found to Be Highly Expressed in HiPSC-Derived Smooth Muscle Cells With the Stroke-Associated Risk Variant

**FIGURE 1:**
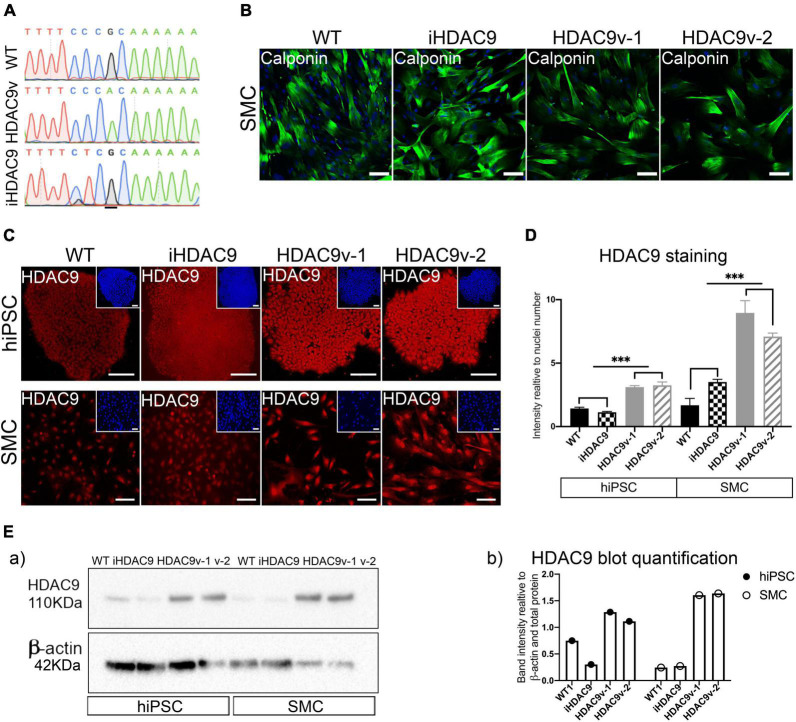
The rs2107596 stroke risk variant correlates with increased HDAC9 expression in two hiPSC derived smooth muscle cells (SMC) lines. **(A)** Representative genomic sequencing of hiPSC for WT, HDAC9 risk variant (HADC9v), and isogenic corrected (iHDAC9) lines. **(B)** hiPSC-derived SMC stained for specific SM marker (Calponin) at mature stage (2 weeks in serum) for WT1, iHDAC9, HDAC9v-1 and HDAC9v-2 lines **(C)** Immunostaining for HDAC9 in hiPSC and SMC for WT, iHDAC9, HDAC9v-1, and HDAC9v-2 lines. Nuclear counterstaining performed with DAPI (insets). **(D)** Quantification of HDAC9 staining relative to cell numbers in hiPSC and SMC stages for WT (*n* = 3), iHDAC9, HDAC9v-1, and HDAC9v-2 lines. **(E)** Cropped blot of HDAC9 protein in WT1, iHDAC9, HDAC9v-1 and HDAC9v-2 SMC (a) and quantification (b). hiPSC = induced pluripotent stem cells; SMC = smooth muscle cells; iHDAC9 = isogenic control; HDAC9v-1 = stroke risk variant line 1; HDAC9v-2 = stroke risk variant line 2. Scale bar = 100 μm. The results are presented as means ± SD of three independent experiments. ***P < 0.001. Statistical analysis was performed by 1-way ANOVA with Tukey’s multiple comparison test.

To assess the effect of *HDAC9* risk variant on vascular cell phenotypes, we compared two hiPSC lines carrying the stroke risk homozygous variant rs2107595 (HDAC9v-1 and HDAC9v-2) in the 3 UTR of *HDAC9* with three wild-type (WT) hiPSC lines from healthy individuals, which were used as controls (Figure 1A and Supplementary Table 1) (14). To understand the dependency of genetic background, we included a further control: a CRISPR-Cas9 isogenic line (iHDAC9) in which the risk nucleotide (A) was replaced by the WT nucleotide (G) (Figure 1A and Supplementary Figures 1A,B).

hiPSC lines were successfully differentiated into vascular smooth muscle cells (SMC), *via* an intermediate neural crest cell population (NC), as previously described (15, 16). hiPSC and NC were characterized for pluripotency and neural crest specific markers, respectively by immunostaining and RT-qPCR analysis (Supplementary Figures 1C–F). The fully differentiated NC-derived SMC were characterized for the expression of SM markers, including calponin (*CNN1*), SM-α-actin (*ACTA2*), and SM22 (*TAGLN*), at both protein and mRNA levels (Figure 1B and Supplementary Figure 1G).

Histone deacetylase 9 expression was analyzed during differentiation by immunostaining and increased protein levels for HDAC9 were seen exclusively in HDAC9v-1 and HDAC9v-2 derived hiPSC and SMC compared to the WT controls and isogenic iHDAC9 lines (Figures 1C,D). This was also confirmed by western blotting analysis (Figure 1E). In contrast, the expression of the neighbor gene, *TWIST*, which is located 150 kb downstream *HDAC9* 3′ end, was found not to be affected in the lines harboring the risk SNP (Supplementary Figure 2A). This observation is consistent with previous findings, which showed that the rs2107595 risk allele exhibited higher transcriptional capacity *via* direct physical interaction between the rs2107595 region and the *HDAC9* promoter (17). Interestingly, we did not observe changes in HDAC9 expression levels in NC intermediates and endothelial cells (EC) generated from WT, iHDAC9, HDAC9v-1 and HDAC9v-2 hiPSC lines (18) (Supplementary Figures 2B,C).

### Histone Deacetylase 9 Stroke Risk Smooth Muscle Cells Proliferation, Migration, and Apoptosis Phenotypes Are Rescued by Sodium Valproate and Small Molecules Inhibitors

**FIGURE 2:**
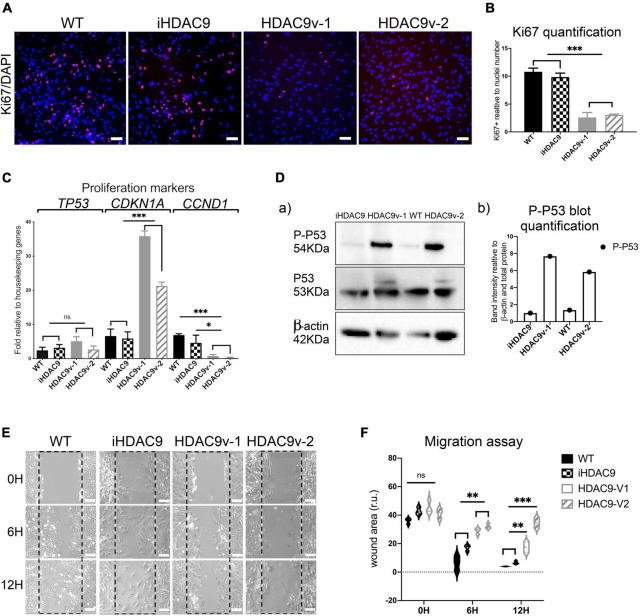
Risk variant *HDAC9* hiPSC-derived SMC show lower proliferative rate and migration ability. **(A)** Ki67 staining was used to measure the proliferation rate of WT1, iHDAC9, HDAC9v-1 and HDAC9v-2 SMC and nuclear counterstaining was performed with DAPI. **(B)** Quantification of Ki67 staining was expressed relative to cells number (nuclei). **(C)** mRNA expression analysis of *TP53* (P53) *CDKN1A* (p21), *CCND1* (CYCLIN D1) in WT (*n* = 3), iHDAC9, HDAC9v-1, and HDAC9v-2 SMC. **(D)** Cropped blot of phospho-p53 (P-P53), total P53 and β-actin control for WT1, iHDAC9, HDAC9v-1 and HDAC9v-2 SMC (a) and quantification (b); **(E)** Representative images of scratch wound assays for WT1, iHDAC9, HDAC9v-1 and HDAC9-v2 SMC lines at the indicated time points (0, 6, and 12 h), and **(F)** quantification of the wound areas. Scale bar = 100 μm. The results are presented as means ± SD of three independent experiments. *P < 0.05; **P < 0.01; ***P < 0.001; ns, not significant. Statistical analysis was performed by 1-way **(B,C)** and 2-way **(F)** ANOVA with Tukey’s multiple comparison test.

Smooth muscle cells derived from HDAC9 risk variant lines derived showed a marked decrease in their proliferative capacity, as shown by low ki67 levels, compared to WT and iHDAC9 control lines (Figures 2A,B). Reduced proliferation was associated with enhanced P21(*CDKN1A*), senescent-cell-cycle marker, and decreased CyclinD1 (*CCND1*) mRNA expression levels (Figure 2C). While a modest increase of p53 (*TP53*) was observed by RT-qPCR, a marked increase of P53-serine 15 phosphorylation (P-P53), which is required for P53 functions in cell cycle arrest and cell death programme, was detected by western blotting in HDAC9v-1 and HDAC9v-2 SMC compared to WT1 and isogenic SMC lines (Figure 2D). Moreover, HDAC9v-1 and -v2 SMC showed reduced migration ability compared to iHDAC9 and WT controls (Figures 2E,F).

**FIGURE 3:**
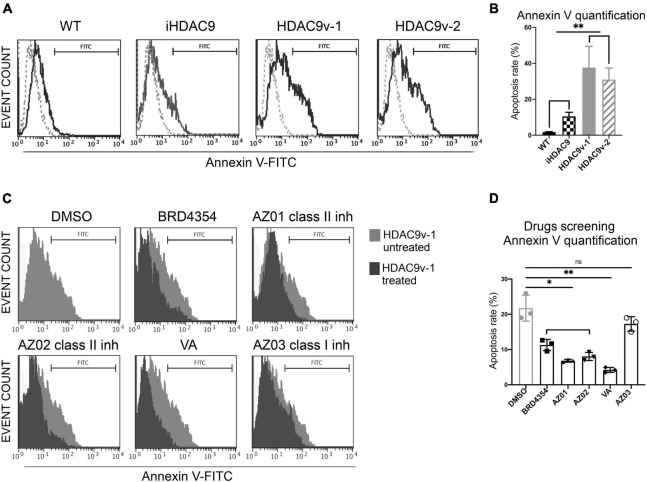
Smooth muscle cells with the stroke risk variant show higher level of apoptosis and respond to HDAC inhibitors treatment. **(A)** Flow cytometric analysis of Annexin V (FITC) show percentage gated for in WT1 (2.9%), iHDAC9 (9.6%), HDAC9v-1 (27.64%), and HDAC9v-2 (25.74%) SMC and unstained (dotted lines) and **(B)** Annexin V staining quantification (*n* = 3). **(C)** SMC flow cytometric analysis of Annexin V (FITC) show percentage gated for HDAC9v-1 untreated (DMSO; 25.92%) and upon treatment with BRD4354 (HDAC9/HDAC5 inhibitor; 7.2%) (10 μmol/L); AstraZeneca HDAC class II inhibitors AZ01 (3.52%) and AZ02 (4.30%) (10 μmol/L); sodium valproate [valproic acid (VA); 3%] (1 mmol/L); and HDAC class I inhibitor AZ03 (19.81%) (10 μmol/L); and **(D)** Annexin V positive staining quantification (*n* = 3). The results are presented as means ± SD of three independent experiments. *P < 0.05; **P < 0.01; ns, not significant. Statistical analysis was performed by 1-way ANOVA with Tukey’s multiple comparison test.

Remarkably, HDAC9 risk lines derived SMC showed significantly higher levels of apoptosis detected by flow cytometric staining for Annexin V compared to WT, and iHDAC9 SMC (Figures 3A,B). This observation was confirmed by flow cytometric assay for caspase-3 and -7, which show higher caspase activity and higher cell death by propidium iodide (PI) staining for the risk HDAC9 SMC compared to the controls (Supplementary Figure 3). Based on these observations, we set up a phenotypic drug screen, by comparing the efficacy of the HDAC pan-inhibitor sodium valproate (VA), BRD4354, a zinc chelating reversible inhibitor with selectivity for HDAC5 and HDAC9, two HDAC class II inhibitors (AZ01 and AZ02) and a HDAC class I inhibitor (AZ03) kindly provided by AstraZeneca (Figures 3C,D). All class II inhibitors appeared to have potential therapeutic efficacy, with VA appearing to be more effective in reducing apoptotic events. In contrast, HDAC class I inhibitors had little effect in reversing apoptosis.

**FIGURE 4:**
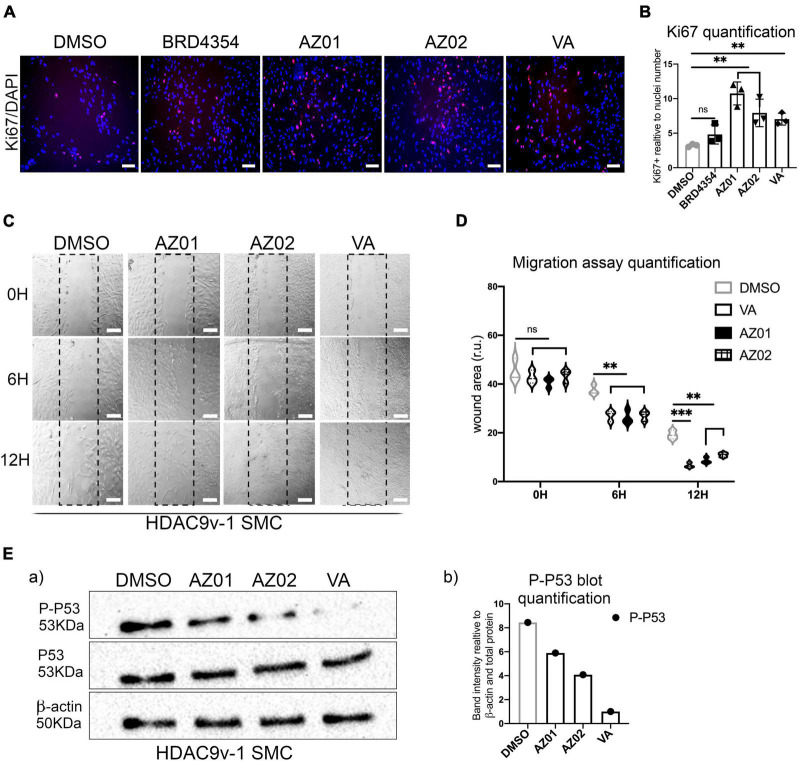
Valproate and AZ class II inhibitors rescue risk SMC proliferation and migration phenotypes. **(A)** Proliferation rate measured by Ki67 staining in HDAC9-v1 control (DMSO) and treated with BRD4354, AZ01, AZ02 HDAC class II inhibitors and sodium valproate (VA) and **(B)** quantification of Ki67 staining expressed relative to cells number (nuclei). **(C)** Representative images of scratch wound assays of iHDAC9 and HDAC9v-1 SMC treated with AZ01, AZ02, and VA at the indicated time points (0, 6, and 12 h) and **(D)** quantification of the wound areas. **(E)** Cropped blot of phospho-p53 (P-P53), total P53 and β-actin control for HDAC9v-1 control (DMSO) and upon treatment with AZ01, AZ02 and VA (a) and quantification (b). Nuclei were stained with DAPI. Scale bar = 100 μm. The results are presented as means ± SD of three independent experiments. **P < 0.01; ***P < 0.001; ns, not significant. Statistical analysis was performed by 1-way **(B)** and 2-way **(D)** ANOVA with Tukey’s multiple comparison test.

Moreover, VA and AZ HDAC class II inhibitors AZ01 and AZ02 were able to rescue SMC proliferation (Figures 4A,B). BRD4354 was not effective in rescuing the proliferation rate in the risk line and was not used in the subsequent rescue experiments. Treatments with AZ01, AZ02, and VA appear also to improve the migration rate in HDAC9-v1 SMC to levels comparable to the isogenic control (Figures 4C,D). Moreover P-P53 levels were seen reduced upon treatment with AZ01, AZ02 and VA HDAC inhibitors in HDAC9v-1 SMC compared to the control (DMSO) (Figure 4E).

### Histone Deacetylase 9 Stroke Risk Smooth Muscle Cells Show Increased NF-κB Mediated ICAM-1 Response and High IL-1β Levels Upon TNF-α Stimulation

**FIGURE 5:**
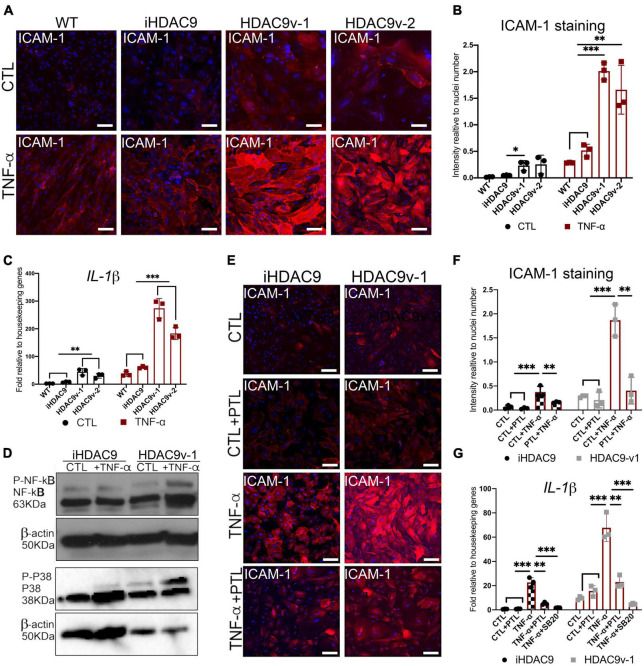
The *HDAC9* risk variant promotes inflammation in SMC through NF-κB activation. **(A)** Cell surface ICAM-1 immunostaining in WT, iHDAC9, HDAC9v-1, and HDAC9v-2 SMC control (CTL) and upon TNF-α stimulation for 24 h (TNF-α) and **(B)** quantification of ICAM-1 staining relative to cells number (nuclei). **(C)** RT-qPCR profile for *IL-1*β in WT (*n* = 3), iHDAC9, HDAC9v-1, and HDAC9v-2 SMC CTL and upon TNF-α treatment. **(D)** Cropped blot of Phospho- NF-κB, total NF-κB, Phospho- p38, total p38, and correspondent β-actin in iHDAC9 and HDAC9v-1SMC CTL and SMC treated with TNF-α (correspondent quantification in Supplementary Figure 4G). **(E)** ICAM-1 immunofluorescence analysis in iHDAC9 and HDAC9v-1 SMC CTL (DMSO), and SMC upon TNF-α stimulation alone and with the NF-κB inhibitor Parthenolide (PTL; 5 μmol/L) and **(F)** ICAM-1 staining quantification relative to cell number (nuclei). **(G)** Expression profile for *IL-1*β in SMC untreated (CTL), treated with PTL or a specific p38 MAPK inhibitor, SB203580 (SB20; 1 μM), with and without TNF-α stimulation. Nuclei were stained with DAPI. Scale bar = 100 μm. The result is representative of three independent experiments (means ± SD). *P < 0.05; **P < 0.01; ***P < 0.001. Statistical analysis was performed by 2-way ANOVA with Tukey’s multiple comparison test.

To test whether HDAC9 promotes inflammatory response in hiPSC-SMC with the stroke risk SNP, SMC were stimulated with the pro-inflammatory cytokine, TNF - α for 30 min, 2, 6, and 24 h. Following this, HDAC9v-1 and v-2 SMC showed a marked increase in the adhesion molecule for leukocytes (ICAM-1) surface expression compared to WT and iHDAC9 lines upon TNF - α treatment, shown here by immunostaining and by flow cytometry (Figures 5A,B and Supplementary Figures 4A–C). To validate the direct effect of *HDAC9* increased expression in promoting inflammatory response, *HDAC9* was efficiently knockdown in iHDAC9 and HDAC9v-1 SMC by gene silencing approach and in turn reduced ICAM-1 surface levels were seen in SMC treated with specific siRNA for HDAC9 compared with control, treated with scramble siRNA (Supplementary Figures 4D–F). We also observed a significant increase in *IL-1*β expression levels in normal state and upon stimulation with TNF-α in the risk variant SMC lines compared to the controls (Figure 5C). It is known that ICAM-1 response upon TNF-α stimulation is mediated by NF-κB activation and recent findings suggest that HDAC9 binds to IKK (inhibitory kappa B kinase), resulting in NF-κB deacetylation and subsequent activation (19). Furthermore, HDAC9 was found to promote MAPK-mediated NF-κB phosphorylation in ischemic stroke (20). Thus, we looked at NF-κB phosphorylation levels in SMC with the risk variant; our results showed a considerable increase in NF-κB Ser536-phosphorylation levels in TNF-α treated HDAC9v-1 SMC sample compared to isogenic control (Figure 5D and Supplementary Figure 4G). Moreover, p38 phospho- levels were found higher in HDAC9v-1 SMC CTL and upon TNF-α treatment in agreement with previous finding suggesting p38 might be regulated by HDAC9 (21) (Figure 5D and Supplementary Figure 4G). Based on these observations, both iHDAC9 and HDAC9v-1 SMC were treated with Parthenolide (PTL; 5 μM), a potent inhibitor of NF-κB activation which acts by specifically inactivating IkappaB kinase. HDAC9-mediated ICAM-1 response to TNF-α stimulation was found to be prevented in SMC treated with PTL compared to control (DMSO) cells (Figures 5E,F). This supports the idea that NF-κB is a key player in the inflammatory response regulated by HDAC9. *IL-1*β expression levels also decrease in SMC treated with PTL and with SB203580 (SB20; 1 μM), a specific p38 MAPK inhibitor, prior to stimulation with TNF-α (Figure 5G). This suggests that p38 might also play an important role in the inflammatory process mediated by HDAC9.

### Histone Deacetylase 9 Stroke Risk Smooth Muscle Cells Show Increased Calcification Markers and Abnormal Intracellular Ca2+ Flux

**FIGURE 6:**
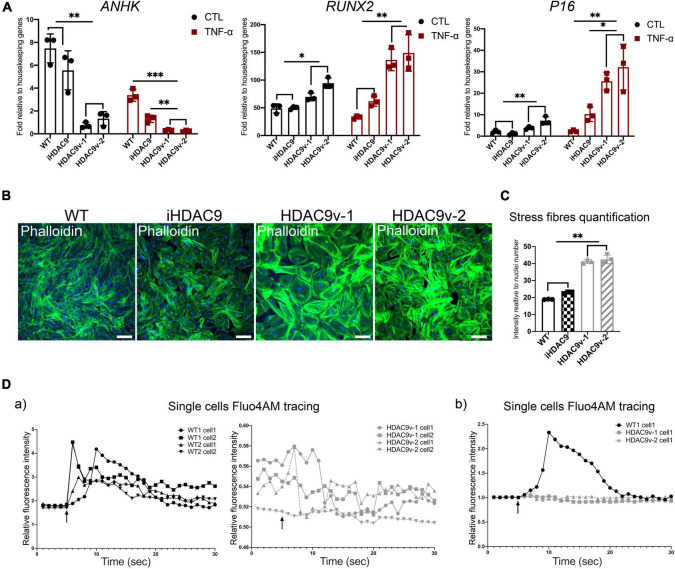
Histone deacetylase 9 risk variant SMC show a senescence-like phenotype. **(A)** RT-qPCR analysis of *ANKH* and *RUNX2* and *P16* genes in WT (*n* = 3), iHDAC9, HDAC9v-1, and HDAC9v-2 CTL and upon TNF-α stimulation. **(B)** Phalloidin staining for WT, iHDAC9, HDAC9v-1 and HDAC9v-2 SMC and **(C)** quantification. **(D)** Single-cell fluorescence tracing of WT1, WT2, HDAC9v-1 and HDAC9v-2 SMC before and after carbachol stimulation (5 s), relative to basal level and total Fluo-4AM loading (a) and compared to WT (b). Nuclei were stained with DAPI. Scale bar = 100 μm. The result is representative of three independent experiments (means ± SD). *P < 0.05; **P < 0.01. Statistical analysis was performed by 2-way **(A)** and 1-way **(C)** ANOVA with Tukey’s multiple comparison test.

It has been shown that NF-κB can promote vascular calcification though the inhibition of ankylosis protein homolog (*ANKH*) expression (22). Consistent with this, we observed decreased *ANKH* mRNA expression levels in HDAC9v-1 and v-2 SMC in both control and TNF-α treated SMC compared to WT, and isogenic cells (Figure 6A). Conversely, *RUNX2*, transcription factor previously associated with arterial calcification, and the senescence marker *P16*, were seen upregulated in the risk SMC lines upon TNF-α stimulation compared to controls (Figure 6A). Upon treatment with TNF-α, *ACTA2* and *TAGLN* expression levels appear to decrease, while IL-1β increases (Figure 5G and Supplementary Figure 4H). This supports the idea that TNF-α inhibits SMC contractile phenotype and induce pro-inflammatory markers.

**FIGURE 7:**
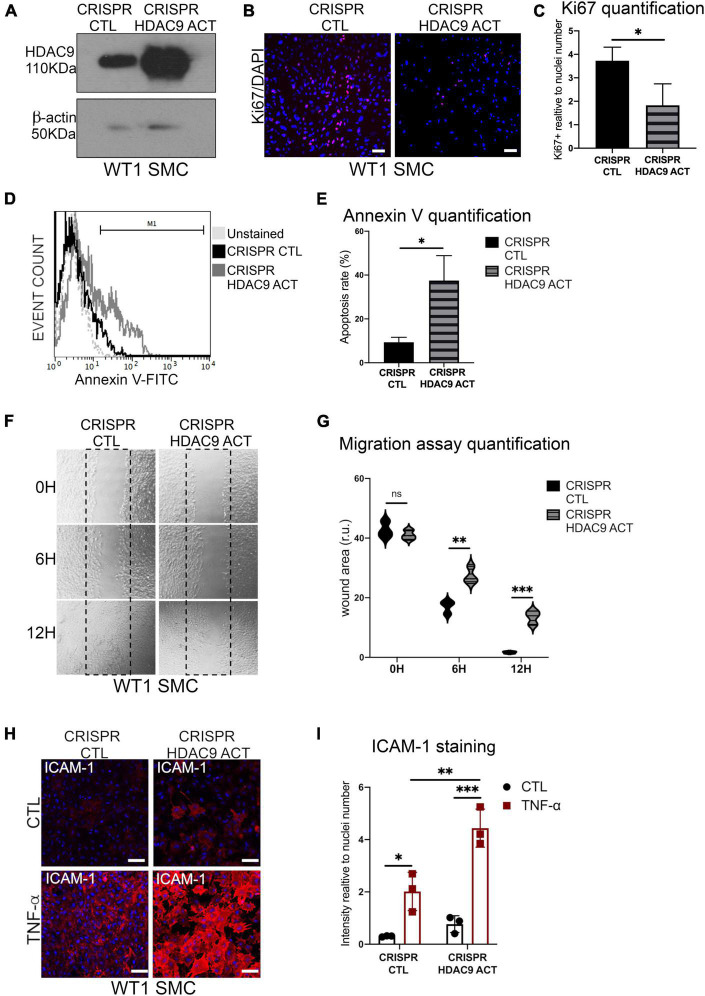
Histone deacetylase 9 overexpression drives SMC phenotypes. **(A)** Cropped blot of HDAC9 and β-actin in WT1 hiPSC-derived SMC transfected with control CRISPR Plasmid (CRISPR CTL) and HDAC9 CRISPR Activation plasmid (CRISPR HDAC9 ACT). **(B)** Ki67 proliferation staining in CRISPR activation plasmid transfected control (CRISPR CTL) and HDAC9 (CRISPR HDAC9 ACT) SMC; and **(C)** quantification of Ki67 staining expressed relative to cells number (nuclei). **(D)** Flow cytometric analysis of Annexin V (FITC) show percentage gated for in CRISPR CTL (12.9%) and CRISPR HDAC9 ACT (33.6%), SMC and unstained (dotted lines) and **(E)** Annexin V staining quantification. **(F)** Representative images of scratch wound assays for CRISPR activation transfected control (CRISPR CTL) and HDAC9 (CRISPR HDAC9 ACT) WT1 hiPSC-derived SMC at the indicated time points (0, 6, and 12 h) and **(G)** quantification of the wound areas. **(H)** Cell surface ICAM-1 immunostaining in CRISPR CTL and CRISPR HDAC9 ACT WT1 SMC control (CTL) and upon TNF-α stimulation for 24 h (TNF-α), and **(I)** quantification of ICAM-1 staining relative to cells number (nuclei). Nuclei were stained with DAPI. Scale bar = 100 μm. The results are presented as means ± SD of three independent experiments. *P < 0.05; **P < 0.01; ***P < 0.001; ns, not significant. Statistical analysis was performed by unpaired Student’s *t*-test **(C,E)** and by 2-way ANOVA **(G,I)** ANOVA with Tukey’s multiple comparison test.

Moreover, HDAC9v-1 and v-2 SMC showed a higher density of stress fibers by phalloidin staining compared to WT and iHDAC9 SMC, suggestive of vascular stiffness (Figures 6B,C). Since intracellular calcium regulates SMC contractility, we also measured calcium flux in response to the cholinergic agonist carbachol as a proxy for contraction. Interestingly, calcium flux appeared to be greatly compromised in HDAC9 risk variant SMC lines compared to WT cells (Figure 6D). To confirm that the phenotypic changes observed in HDAC9v-1 and -v2 risk lines are driven by *HDAC9* increased expression, WT1 hiPSC-derived SMC were transfected with either a control CRISPR vector (CRISPR CTL) or a HDAC9 CRISPR Activation plasmid (CRISPR HDAC9 ACT) to increase gene expression, which was detected by western blotting analysis (Figure 7A). Transfected WT1 SMC were analyzed for proliferation by ki67 staining (Figures 7B–C), apoptosis by Annexin V staining (Figures 7D–E), migration by scratch assay (Figures 7F–G) and ICAM-1 surface staining upon TNF-alpha treatment (Figures 7H–I). Activated SMC show lower proliferation rate and migration ability, increase cell death and ICAM-1 response upon TNF-alpha stimulation similar to what we observed in HDAC9 risk lines. Taken together, these findings suggest that increased *HDAC9* expression observed in the stroke risk variant lines could be implicated in the altered regulation of SMC functions, including proliferation, migration, inflammation and contraction.

## Discussion

In this study, we developed a relevant hiPSC-derived SMC model to assess the phenotypic effect of the most strongly associated GWAS hit for ischemic stroke in the *HDAC9* gene, and to test HDAC inhibitors for potential therapeutic interventions. HDAC9 is the strongest genetic risk factor for large vessel stroke identified to date. The risk allele (A) in HDAC9 gene has a frequency of 20% across all ethnicities with 42% increased risk of large artery stroke for 1 copy and a 102% increase in risk for both copies of the risk allele.

Our findings provide validation of the importance of HDAC9 in regulating vascular SMC phenotypic changes. Additionally, our findings highlight the potential of HDAC9 inhibition as a therapeutic intervention to potentially prevent progression of large artery stroke, and provide a cellular human hiPSC-derived disease model with easy phenotypic readout for high-throughput screening of such inhibitors. This drug screening method is likely to be applicable to evaluate and screen for therapeutic interventions, for the other many GWAS associations recently identified as risk factors for ischemic stroke (1).

In our experiments we show that the rs2107595 variant is associated with elevated expression of *HDAC9* in vascular SMC. Our findings are supported by the previously identified physical interaction of the rs2107595 region with the HDAC9 promoter, which provides a mechanistic link between genetic variation at rs2107595 and increase *HDAC9* expression by preventing preferential binding of the E2F3/TFDP1/Rb1 repressor complex to the common allele (17). Our results also revealed a cell dependent effect of rs2107595 on HDAC9 expression, since higher *HDAC9* expression levels were observed in hiPSC and fully differentiated vascular SMC, but not in the NC intermediate population nor in hiPSC-derived ECs. We hypothesize that this might be due to a cell-type-specific chromatin conformation affecting the accessibility of the E2F3 transcription complex and thus gene expression.

Our findings support the potential role of HDAC9 in regulating the SMC phenotype, *via* deacetylation of non-histone proteins, which act on other substrates leading to both upregulation and downregulation of genes critical for human development and disease. For example, HDAC9 was shown to deacetylate Lys116 on ATDC (TRIM29; P53 antagonist), preventing ATDC from binding P53, and consequently leading to activation of P53-inducible genes in mouse fibroblast (23). Our results support the potential role of HDAC9 in regulating P53 signal in SMC by showing that risk lines show an increase in phospho-levels for P53.

Previous studies have implicated inflammation as a possible pathway mediating the effect of HDAC9 on atherosclerosis (12, 24). In support of this, our results show that HDAC9 promotes expression of ICAM-1 on the surface of SMC in response to TNF-α stimulation, by enhancing NF-κB activation, a key factor in atherogenesis and atheroprogression (27). Notably, upstream inhibition of NF-κB in our model was able to prevent HDAC9-mediated SMC inflammatory response, suggesting that NF-κB could be a key factor in HDAC9-driven pathogenesis, as previously suggested (19, 20). However, given the widespread expression of HDAC9 in multiple cell types, including in macrophage and T-cells, which play an important role in disease progression, we cannot exclude that in addition to SMC, other cells could be affected by the *HDAC9* risk variant, and therefore further investigations in these cell types is required.

Another important limitation of our model is that it does not include the conventional risk factors, such as high blood pressure, cholesterol and diabetes, which are important in stroke risk. However, a substantial proportion of stroke risk remains unexplained and there is strong evidence that the genetic component is important too. Our hiPSC model may help us to understand the unexplained stroke risk by exploring the genetic driven underlying mechanisms.

We have previously demonstrated that human iPSC have the potential to reproduce *in vitro* vascular disease relevant phenotypes and mechanisms that can be chemically targeted in a controlled environment (25). However, our *HDAC9* SMC model is the first example of “functional genomic” applied to stroke associated risk variants identified by GWAS studies and opens the way to dissect the causal role of these single SNPs on molecular and cellular phenotypes in appropriate human cells.

Finally, the disease-relevant phenotype observed in our hiPSC-derived SMC model and the easily detectable phenotypic readout makes it an attractive platform for the screening of potential inhibitors as demonstrated by our small-scale test. In our system, we compared a number of HDAC inhibitors and found that the antiepileptic drug sodium valproate (VA), a pan-HDAC inhibitory, was the most effective in reducing our read-out of cell death in SMC with the risk variant to levels comparable to healthy control. Furthermore, VA was able to recue phenotypic changes, including proliferation and migration. However, VA has multiple other non-HDAC inhibitory effects as well as significant side-effects, and therefore specific inhibitors for HDAC9 and/or identification of drugable downstream target(s) may be more therapeutically effective, and our model provides a screening system in which their efficacy could be evaluated.

## Materials and Methods

### HiPSC Culture

Wild-type and HDAC9 rs9107595 risk hiPSC lines were purchased from the HiPSci Human stem cell initiative cell bank or obtained from the hiPSC core facilities at Cambridge (Supplementary Table 1)^1^ ^,2^ and cultured in TeSR-E8 media (STEMCELL Technologies, Vancouver, BC, Canada) or E8 media (Dulbecco’s Modified Eagle Medium/Nutrient Mixture F-12 (DMEM/F-12) with Insulin-Transferrin-Selenium (Thermo Fisher Scientific, Waltham, MA, United States), Sodium Bicarbonate (Thermo Fisher Scientific, Waltham, MA, United States), and L-ascorbic acid (Merck) supplemented with FGF2 (4 ug/mL; Biochemistry Department, University of Cambridge) and TGF- β1 (1.74 ug/mL; R&D Systems, Minneapolis, MN, United States) using Vitronectin XF (STEMCELL Technologies, Vancouver, BC, Canada) as chemically defined xenofree cell culture matrix. All hiPSC lines were validated by Cambridge Biomedical Research Center iPS Core and routinely tested for presence of mycoplasma contamination by Mycoplasma Experience Ltd.

### HiPSC Differentiation Into Neural Crest Cell Population-Smooth Muscle Cells

For NC differentiation, hiPSC were detached from Vitronectin coated plates using ReLeSR (STEMCELL Technologies, Vancouver, BC, Canada). Clumps were plated at a density of 300 in 0.1% gelatin-coated six well plates in CDM-polyvinyl alcohol (PVA) for 4 days without splitting. CDM was composed of Iscove’s modified Dulbecco’s medium plus Ham’s F12 NUT-MIX (Thermo Fisher Scientific, Waltham, MA, United States) medium in a 1:1 ratio, supplemented with chemically defined lipid concentrate (Thermo Fisher Scientific, Waltham, MA, United States), transferrin (Roche Diagnostics, Basel, Switzerland), insulin (Roche Diagnostics, Basel, Switzerland), and monothioglycerol (Sigma) supplemented with FGF2 (12 ng/mL; R&D Systems, Minneapolis, MN, United States) and SB-431542 (10 mmol/L; Tocris), referred as FSB. After 4 days, hiPSC was dissociated using TrypLE Express (Gibco) and seeded as single cells at a 1:3 ratio on 0.1% gelatin-coated plates in FSB. NC cells were passaged every time reached confluence, up to 12 passages.

For NC-SMC differentiation, NC cells were dissociated using TrypLE Express and cultured in SMC differentiation medium [CDM-PVA supplemented with PDGF-BB (10 ng/ml, Peprotech) and TGF-β1 (2 ng/ml, Peprotech)] for 12 d. For long-term cultures, SMCs were subsequently grown in MEM (Sigma-Aldrich M5650) containing 10% FBS (Sigma-Aldrich F7524) up to 10 passages.

### HiPSC Differentiation Into Endothelial Cells

For endothelial cells (EC) differentiation, the protocol was adapted from (18). hiPSC were detached from Vitronectin coated plates using ReLeSR. Clumps were plated at a density of 300 colonies/well in 0.1% gelatin-coated six well plates in CDM-polyvinyl alcohol (PVA) supplemented with FGF (20 ng/mL; R&D Systems, Minneapolis, MN, United States), LY294002 (10 mmol/L; MERCK) and BMP4 (10 ng/ml: R&D Systems) for 36 h. Medium was then replaced with StemPro-34 (Gibco) supplemented with VEGF-A (100 ng/ml; Peprotech), Forskolin (2 umol/L; Tocris) and Vitamin C (1 mmol/L, MERCK). After 4 days, hiPSC was dissociated using TrypLE Express (Gibco) and resuspended in PBE [phosphate-buffered saline (PBS), pH 7.2, 0.5% bovine serum albumin (BSA; MERCK), and 2 mM EDTA (MERCK)] for MACS sorting using MiniMACS separator and kit (Miltenyi Biotec). Sorting was performed using CD34 MicroBead kit (Miltenyi Biotec) and sorted cells were resuspended up to 10^8^ in 500 ul PBE buffer. Cells were then plated in 0.1% gelatin-coated six well plates with StemPro-34 supplemented with VEGF-A (50 ng/ml) and FGF (20 ng/ml) for analyses.

### CRISPR-Mediated Gene Editing

#### CRISPR-Cas9–Based HDAC9 Correction

The isogenic (iHDAC9) line was originated from HDAC9v-1 line using single guide synthetic RNA (sgRNA; Synthego), SpCas9 protein (Biochemistry Department, University of Cambridge, Cambridge, United Kingdom), and a 90-nt single-stranded oligodeoxynucleotide (ssODN; IDT) for homology-directed repair (29) (Supplementary Table 2). To avoid ssODN cleavage by Cas9, a silent mutation was introduced in the NGG codon upstream of the correction site (Supplementary Figures 1A,B). For gene targeting, 2,00,000 cells were electroporated with Cas9/sgRNA together with ssODN using the Amaxa 4DNucleofector CA-137 program code (Lonza). Transfected cells were plated onto vitronectin coated-plates in TeSR-E8 with Y-27632 (10 mmol/L) and CloneR (STEMCELL Technologies, Vancouver, BC, Canada). After 48 h, the pool of transfected cells was sequenced to test recombination efficiency. Positive clones were selected by serial dilution and manual selection.

### Quantitative Real-Time Polymerase Chain Reaction

Complementary DNA (cDNA) was synthesized from 250 ng total RNA using the Maxima First Strand cDNA Synthesis Kit (Thermo Fisher Scientific, Waltham, MA, United States). Quantitative realtime polymerase chain reaction (qRT-PCR) mixtures were prepared with the FAST-SYBR Green Master Mix (Thermo Fisher Scientific, Waltham, MA, United States) and analyzed using the QuantStudio 7 Flex (Applied Biosystems, Thermo Fisher, Waltham, MA, United States). CT values were normalized to housekeeping genes, GAPDH and PDGB using standard curve system. Primer sequences are listed in Supplementary Table 3.

### HDAC9 CRIPSR Activation Plasmid Transfection and Selection

WT1 hiPSC-derived SMC were seeded in a 6-well tissue culture plate and grow to 80% cell confluency prior to transfection. SMC were transfected with 1 ug of HDAC9 CRISPR Activation plasmid (sc-401419-ACT) or with the control CRISPR Activation Plasmid (sc-437275) within 10 ul of UltraCruz Transfection Reagent according to manufacturer’s protocol. After 24 h, positively transfected SMC were selected *via* Puromycin dihydrochloride selection (10 ug/ml; sc-108071). Positive selected cells were analyzed 72 h after transfection step for HDAC9 protein levels by western blotting as described below.

### Western Blotting

Cells were lysed in RIPA buffer with added phosphatase inhibitor cocktail (Sigma) and protease inhibitor cocktail (Sigma) on ice for 15 min. Protein content was quantified by Pierce Bicinchoninic Acid (BCA) Protein Assay Kit (Thermo Fisher Scientific, Waltham, MA, United States). Samples was resolved by electrophoresis on 10–12% Tris–HCl precast sodium dodecyl sulfate (SDS)-polyacrylamide gel (Bio-Rad), then transferred to polyvinylidine difluoride membranes (PVDF; Millipore). Membranes were blocked for 1 h at room temperature with 5% BSA in Tris-Buffered Saline containing 0.1% Tween-20 (TBS-T; Sigma) and incubated overnight with primary antibodies (Supplementary Table 4) at 4°C. Membranes were washed with TBS-T, incubated with horseradish peroxidase (HRP)-conjugated secondary antibodies for 1 h at room temperature and developed with the Pierce ECL2 western blotting substrate (Thermo Fisher Scientific, Waltham, MA, United States) using Gel Doc™ XR + system (BioRad). The ImageLab™ Software (v5.2, BioRad) High Resolution programme with Signal Accumulation Mode was used to capture images at incremental exposure times. Anti-β-actin antibody was used as control for equal loading and transfer of the samples. Full-length blots are shown in Supplementary Table 5.

### Immunofluorescence Staining of HiPSC, Neural Crest Cell Population, Smooth Muscle Cells, and Endothelial Cells

Adherent cells were fixed using 4% PFA, permeabilized with 0.5% Triton X-100 in PBS (Sigma), and blocked with PBS + 3% BSA for 60 min at room temperature. For the detection of cell surface ICAM-1, NC-SMC were incubated in blocking solution without permeabilization. For cytoskeletal analysis, cells were stained with CytoPainter Phalloidin-iFluor 488 (1:200; Abcam). Primary antibodies (Supplementary Table 4) incubations were performed at 4°C overnight and Alexa Fluor tagged secondary antibodies (1:500, Molecular Probes Invitrogen) applied for 1 h at room temperature the following day. Images were acquired on a Zeiss LSM 700 confocal and Leica TCS SP5 microscopes and analyzed with Fiji-ImageJ software. Five images were taken for each well of each in three independent experiments done for each condition.

### Flow Cytometry Assays

#### Annexin V Apoptosis Assay

1 × 106 cells/ml were harvested and resuspended in 1 × annexin-binding buffer and incubated with 5 μl of Annexin V–488 (Alexa Fluor 488 Annexin V/Dead Cell Apoptosis Kit; Life technologies, Carlsbad, CA, United States) for 15 min at room temperature. Cells were then resuspended in PBS and measured with a BD LRSFortessa Flow cytometer. Flow cytometric data were analyzed with FCSalyzer 0.9.15-alpha software.

#### Caspases Assay

Smooth muscle cells were treated for caspases activity using the Vybrant^®^ FAM Caspase-3 and -7 Assay kit (V35118; Life technologies, Carlsbad, CA, United States) according to manufacture instructors. As a positive control, WT1 SMC were treated with 0.5 uM Staurosporine for 3 h. Cells were trypsinised and the resuspension was incubated with FAM-488 for 1 h at 37 degrees. After two washes, cells were incubated with propidium idodide (PI; 10 μg/ml) to visualize dead cells. Cells were then resuspended in PBS and measured with BD LRSFortessa Flow cytometer. Flow cytometric data was analyzed with FlowJo VX software.

### TNF-α and Drug Treatment

Cells were treated with tumor necrosis factor TNF-α (10 ng/ml; Peprotech) in DMEM + 10% FBS for 30 min, 2, 6, and 24 h and then harvested for analyses. SMC were treated with sodium valproate (1 mmol/L; Merck), BRD4354 (1 uM; Tocris), AstraZeneca compounds (HDAC class II inhibitors AZ01, AZ02 and class I inhibitor AZ03; 10 uM), Parthenolide (5 μM; Merck) and SB203580 (5 μM; Merck) or transfected with ON-TARGETplus siRNA for Human HDAC9 (Dharmacon; 20 nM) in DMEM + 10% FBS 4 days prior to be harvested for analyses.

### Cell Proliferation Assay

To assess cell proliferation in fixed cells, ki67 staining (Cell Signaling Technology) was performed overnight at 4°C. The following day, the cells were incubated with a secondary Alexa 548 tagged antibody (Molecular Probes Invitrogen) and DAPI for 1 h at room temperature. The numbers of ki67-positive and the total number of cells (DAPI-stained nuclei) were counted with the Cell Counter Plugin in Fiji/ImageJ.

### Scratch Migration Assay

Cells were plated onto 12-well plates and allowed to form a confluent monolayer. The cell monolayer was then scratched in a straight line to make a “scratch wound” with a 1-mL pipette tip. Cells were maintained in DMEM and images of the closure of the scratch were captured at different time points as indicated. Cells were tracked using the Wound_healing_size_tool macro for Fiji/ImageJ.

### Calcium Fluo4AM Contraction Assay

Smooth muscle cells were preloaded with the calcium-sensitive fluorophore Fluo4AM (2.5 μM, Molecular Probes) in normal extracellular solution (NES; 140 mmol/L NaCl, 5 mmol/L KCl, 2 mmol/L CaCl2, 1 mmol/L MgCl2, 10 mmol/L glucose and 10 mmol/L HEPES, pH 7.3) for 1 h at room temperature. Cells were the washed at room temperature. Intracellular calcium flux was monitored as time series with acquisition rates of 1 frame every 0.2 ms over 1 min using a Zeiss LSM 700 confocal microscope before and after addition of carbachol (100 μM, Sigma-Aldrich). Total Fluo4AM uptake was measured by treating the cells with 0.1% Triton X-100 to permeabilize the membranes. For experiment, five cells were randomly picked from a field of view, and the fluorescent trace was analyzed using Fiji/ImageJ software.

### Statistical Analysis

Statistical analyses were performed using GraphPad Prism 9 software. One-way and Two-way ANOVA with Tukey’s multiple comparison test was used for the analysis of three or more groups. Unpaired Student’s *t*-test was to determine statistically significant differences between two groups. Results are presented as standard deviation (SD). All experiments represent the results of at least three independent biological replicates (measurements of biologically distinct samples). *P < 0.05; **P < 0.01; ***P < 0.001; ****P < 0.0001.

## Data Availability Statement

The raw data supporting the conclusions of this article will be made available by the authors, without undue reservation.

## Author Contributions

AG helped with conception, design, acquisition, analysis, and interpretation of iPSC-derived SMC data, and drafted the manuscript. IK helped with differentiation of SMC, caspases activity, and migration assays. FS and JC conceived and design of the CRISPR-Cas9 strategy. MT contributed with the identification of HDAC9 risk variant lines. SS contributed with the revision of the manuscript. HM helped with obtaining funding, with the revision of themanuscript, and supervision of all studies. All authors contributed to the article and approved the submitted version.

## Author Disclaimer

The views expressed are those of the author(s) and not necessarily those of the NIHR or the Department of Health and Social Care.

## Conflict of Interest

The authors declare that the research was conducted in the absence of any commercial or financial relationships that could be construed as a potential conflict of interest.

## Publisher’s Note

All claims expressed in this article are solely those of the authors and do not necessarily represent those of their affiliated organizations, or those of the publisher, the editors and the reviewers. Any product that may be evaluated in this article, or claim that may be made by its manufacturer, is not guaranteed or endorsed by the publisher.

## Footnotes

1
https://www.hipsci.org

2
https://cambridgebrc.nihr.ac.uk/expandables/hipsc-core-facility/
