# Supplementary material for: The Histone Deacetylase 9 Stroke-Risk Variant Promotes Apoptosis and Inflammation in a Human iPSC-Derived Smooth Muscle Cells Model
Source: Front Cardiovasc Med. 2022 Mar 30;9:849664. doi: 10.3389/fcvm.2022.849664 (PMC9005977; doi:10.3389/fcvm.2022.849664)
Supplement: Supplementary file 1 [file Data_Sheet_1.docx]

Supplementary Material

# Supplementary Figures and Tables

**Supplementary Table 1. iPSC lines**

| Name | Vendor or Source | Sex | URL and Reference |
| --- | --- | --- | --- |
| WT1 (BBHX8) | iPS Core Facility, Cambridge | Male | ref 14; https://cambridgebrc.nihr.ac.uk/expandables/hipsc-core-facility/ |
| WT2 (HPSI0314i-sojd_3) | HIPSCI Consortium | Female | https://www.hipsci.org/lines/#/lines/HPSI0314i-sojd_3 |
| WT3 (HPSI0214i-wibj_2) | HIPSCI Consortium | Female | https://www.hipsci.org/lines/#/lines/HPSI0214i-wibj_2 |
| HDAC9v-1 (CRL) | hiPSC source | Male | https://cambridgebrc.nihr.ac.uk/expandables/hipsc-core-facility/ |
| HDAC9v-2 (HPSI0314i-hoik_1) | HIPSCI Consortium | Female | https://www.hipsci.org/lines/#/lines/HPSI0314i-hoik_1 |
| iHDAC9 (isogenic HDAC9v-1) | iPS Core Facility, Cambridge | Male | - |

**Supplementary Table 2. CRISPR sgRNA guide and ssODN**

| Primers | Sequence |
| --- | --- |
| HDAC9 CORRECTION GUIDE | 5’-ATGTTTTTGTCCCACAGTAA-3’ TGG |
| HDAC9 CORRECTION ssODN | T*G*A*GAAGGATGAGGAGCCATTACTGTGGGACAAAAACATTTTCAC**G**CAAAAAAGAATGTACAAGCACACAAAAAAGATGTGGGATTT*T*T*A |
| HDAC9 CORRECTION SCREENING PF1 | 5’-ATTGCATCCAGGAACTCA-3’ |
| HDAC9 CORRECTION SCREENING PR1 | 5’-TAATGCTTGGCAGGAACA-3’ |

**Supplementary Table 3. Primers sequences**

| Name | Sense sequence | Antisense sequence |
| --- | --- | --- |
| *OCT3/4* | AGGGCAAGCGATCAAGCA | GGAAAGGGACCGAGGAGTA |
| *SOX2* | CTTTTGCACCCCTCCCATTT | CTTTTGCACCCCTCCCATTT |
| *NANOG* | CATGAGTGTGGATCCAGCTTG | CCTGAATAAGCAGATCCATGG |
| *B3GAT1* | CGACGACGACAACACCTACA | CCTGGTAGCCTCCCTTCACA |
| *P75NTR* | ACAAGACCTCATAGCCAGCAC | CTGTTGGCTCCTTGCTTGTTC |
| *CNN1* | GTCCACCCTCCTGGCTTT | AAACTTGTTGGTGCCCATCT |
| *ACTA2* | CACTGTCAGGAATCCTGTGA | CAAAGCCGGCCTTACAGA |
| *TAGLN* | TCTTTGAAGGCAAAGACATGG | TTATGCTCCTGCGCTTTCTT |
| *CCND1* | CCCGCACGATTTCATTGAAC | AGGGCGGATTGGAAATGAAC |
| *CDKN1A* | TGGAGACTCTCAGGGTCGAAA | GGCGTTTGGAGTGGTAGAAATC |
| *TP53* | TGGCCATCTACAAGCAGTCA | GGTACAGTCAGAGCCAACCT |
| *IL-1β* | TGAGCTCGCCAGTGAAATGA | CATGGCCACAACAACTGACG |
| *ANKH* | CAACCTCTTTGTTTCCCGGG | AACAGAGCGTGAGTGACAGA |
| *RUNX2* | CTGTGGTTACTGTCATGGCC | AGGTAGCTACTTGGGGAGGA |
| *P16* | GACCTGGCTGAGGAGCTG | AATCGGGGATGTCTGAGGGA |
| *TWIST* | GCCAGGTACATCGACTTCCTCT | TCCATCCTCCAGACCGAGAAGG |
| *PBGD* | GGAGCCATGTCTGGTAACGG | CCACGCGAATCACTCTCATCT |
| *GAPDH* | AACAGCCTCAAGATCATCAGC | GGATGATGTTCTGGAGAGCC |

**Supplementary Table 4. Primary antibodies**

| Target antigen | Vendor or Sources | Catalog n | Working Concentration | URL |
| --- | --- | --- | --- | --- |
| OCT3/4 | Santa Cruz | sc-5279 | IF 1:100 | https://www.scbt.com/p/oct-3-4-antibody-c-10 |
| SOX2 | R&D | AF2018 | IF 1:300 | https://www.rndsystems.com/products/human-mouse-rat-sox2-antibody_af2018 |
| TRA-1-60 | Santa Cruz | sc-21705 | IF 1:100 | https://www.scbt.com/p/tra-1-60-antibody-tra-1-60 |
| p75 | AbCam | Ab52987 | IF 1:500 | https://www.abcam.com/p75-ngf-receptor-antibody-ep1039y-ab52987.html |
| HNK1 | SIGMA | C6680-100TST | IF 1:100 | https://www.sigmaaldrich.com/GB/en/product/sigma/c6680 |
| Calponin | Sigma | C-2687 | IF 1:10000 | https://www.sigmaaldrich.com/GB/en/product/sigma/c2687 |
| HDAC9 | NOVUS; SIGMA; Abcam | NBP2-03993;HPA028926; ab18970 | WB 1:1000; IF 1:200 | <https://www.novusbio.com/products/hdac9-antibody_nbp2-03993>;  <https://www.sigmaaldrich.com/GB/en/product/sigma/hpa028926>;  https://www.abcam.com/hdac9-antibody-epr5223-ab109446.html |
| P53 | Santa Cruz Biotechnology | sc-126 | WB 1:500 | https://www.scbt.com/p/p53-antibody-do-1 |
| P-P53 (Ser15) | Cell Signaling | 9286T 9284T | WB 1:1000 | https://www.cellsignal.co.uk/products/primary-antibodies/phospho-p53-ser15-16g8-mouse-mab/9286;  https://www.cellsignal.co.uk/products/primary-antibodies/phospho-p53-ser15-antibody/9284 |
| ICAM-1/CD54 | R&D Systems | BBA3 | WB 1:1000; Flow 1:100 | https://www.rndsystems.com/products/human-icam-1-cd54-antibody-bbig-i1-11c81-_bba3 |
| NF-κB p65 | R&D Systems | MAB5078 | WB 1:1000 | https://www.rndsystems.com/products/human-mouse-rat-rela-nfkappab-p65-antibody-532301_mab5078 |
| P-NF-κB p65 | R&D Systems | MAB72261 | WB 1:1000 | https://www.rndsystems.com/products/human-phospho-rela-nfkb-p65-s536-antibody-1091b_mab72261 |
| p38 | Abcam | AB31828 | WB 1:1000 | https://www.abcam.com/p38-deltamapk13--p38-alphamapk14-antibody-m138-ab31828.html |
| P-p38 | Santa Cruz | sc-17852-R | WB 1:1000 | https://www.scbt.com/p/p-p38-antibody-thr-180-tyr-182 |
| Occludin | Thermo Fisher | 331500 | IF 1:200 | https://www.thermofisher.com/antibody/product/Occludin-Antibody-clone-OC-3F10-Monoclonal/33-1500 |
| CytoPainter Phalloidin-iFluor 488 | Abcam | ab176753 | IF 1:200 | https://www.abcam.com/phalloidin-ifluor-488-reagent-ab176753.html |
| Alexa Fluor 488 Annexin V | Life technologies | V13241 | Flow cytometry 1:50 | https://www.thermofisher.com/order/catalog/product/V13241 |
| Vybrant FAM Caspase-3 and -7 Assay | Life technologies | V35118 | Flow cytometry | https://www.thermofisher.com/order/catalog/product/V35117 |
| β-actin | SIGMA | A1978 | WB 1:1000 | https://www.sigmaaldrich.com/GB/en/product/sigma/a1978?context=product |

**
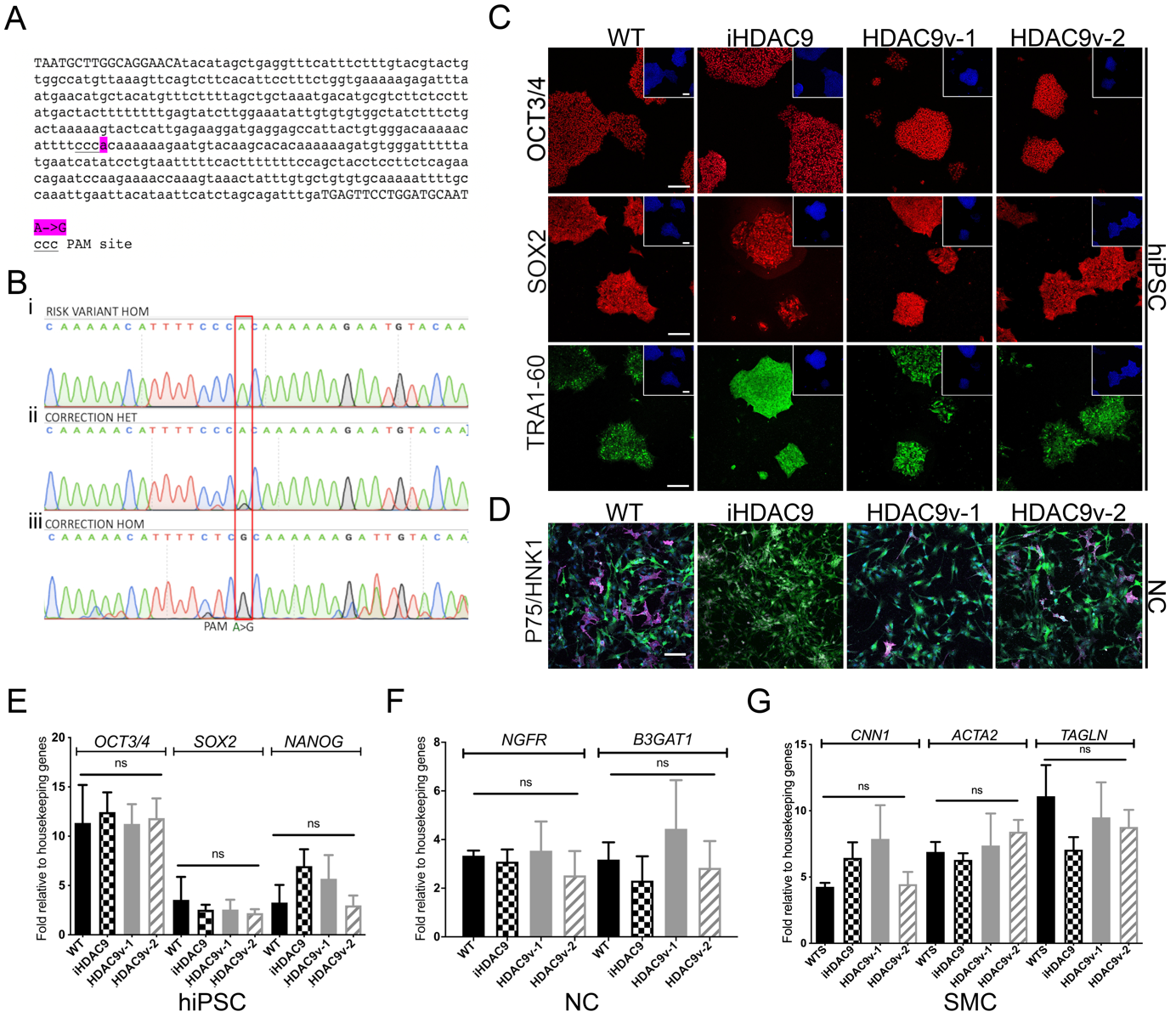
**

**Supplementary Figure 1. Characterization of hiPSC and differentiated SMC lines. A)** Partial sequences of HDAC9 3’ UTR region, with highlighted risk nucleotide (A) and underlined NGG PAM site (CCC). **B)** Sanger sequencing output for **(i)** HDAC9 homozygous risk variant (A) **(ii)** CRISPR/Cas9 targeted pool and **(iii)** homozygous CRISPR corrected clone. **C)** Immunostaining analysis for hiPSC (OCT3/4; SOX2; TRA-1-60) and **(D)** neural crest (NC) p75 (green) and HNK1(magenta) specific markers. Nuclei were stained with DAPI. Scale bar=100μm. **E)** RT-qPCR analysis for pluripotency markers expression (*OCT3/4*, *SOX2* and *NANOG*) and **(F)** for specific markers for the NC intermediate population *NGFR* (P75) and *B3GAT1* (HNK1); **G)** RT-PCR for SMC markers (*CNN1, ACTA2, TGLN*) for WTs (n=3), iHDAC9, HDAC9v-1, and HDAC9v-2 lines. hiPSC= induced pluripotent stem cells; NC=neural crest; SMC=smooth muscle cells; iHDAC9= isogenic control; HDAC9v-1= stroke risk variant line 1; HDAC9v-2= stroke risk variant line 2. The results are presented as means ± SD of three independent experiments; ns, not significant. Statistical analysis was performed by 1-way ANOVA with Tukey’s multiple comparison test.

**
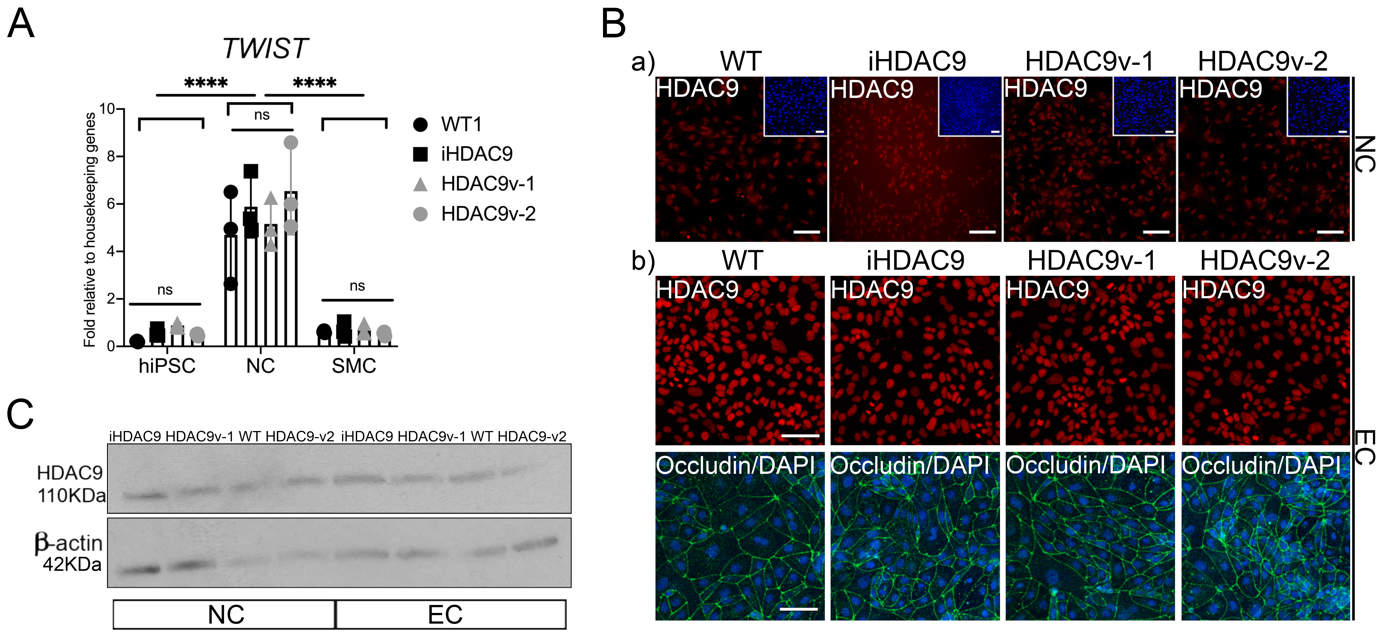
**

**Supplementary Figure 2. TWIST and HDAC9 expression profile in hiPSC-derived cell types. A)** RT-qPCR analysis for *TWIST* expression at hiPSC, NC and SMC stages for WT1, iHDAC9, HDAC9v-1 and HDAC9v-2. **B)** WT, iHDAC9 and risk lines, HDAC9v-1 and -v2, hiPSC-derived NC stained for HDAC9 (**a**) and endothelial cells (EC) stained for HDAC9 the EC marker, Occludin (**b**); nuclei were stained with DAPI. Scale bar=100μm. **C)** Cropped blot of HDAC9 protein in iHDAC9, HDAC9v-1, WT1 and HDAC9v-2 hiPSC-derived NC intermediate population and EC. The results are presented as means ± SD of three independent experiments. *****P* < 0.0001; ns, not significant. Statistical analysis was performed by 1-way ANOVA with Tukey’s multiple comparison test.

**
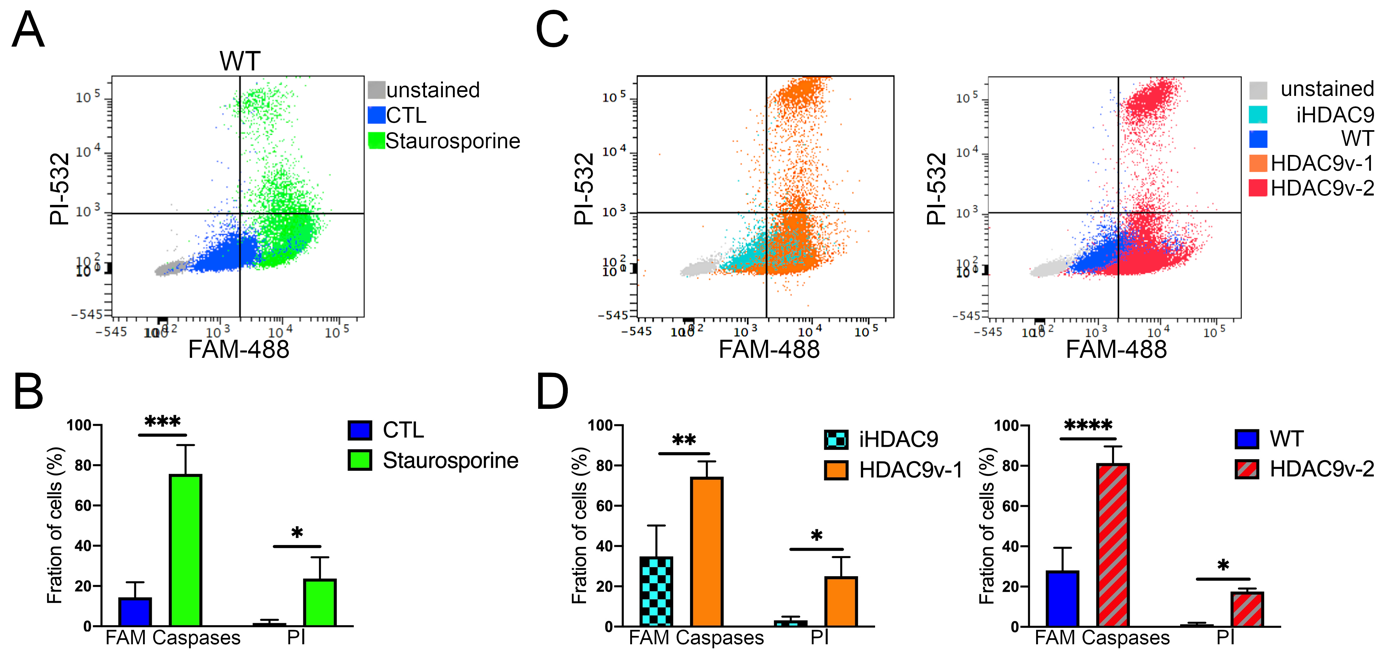
**

**Supplementary Figure 3. Caspases activity in stroke risk and control SMC lines. A)** Apoptotic pathway activation measured by FAM caspase-3 and -7 (FAM-488) and propidium iodide (PI-532) flow cytometry in WT SMC in untreated (control) and treated with Staurosporine (caspase-dependent apoptosis inducer; 0.5uM) conditions was used as positive control and **(B)** quantification. **C)**  FAM caspase assay and PI staining for iHDAC9, WT1, HDAC9v-1 and HDAC9v-2 and **(D)** quantification of the percentage of caspase activity and cell death. The result is representative of three independent experiments (means ± SD). ***P* < 0.01; ****P* < 0.001; *****P* < 0.0001. Statistical analysis was performed by 2-way (B) and 1-way (D) ANOVA with Tukey’s multiple comparison test.


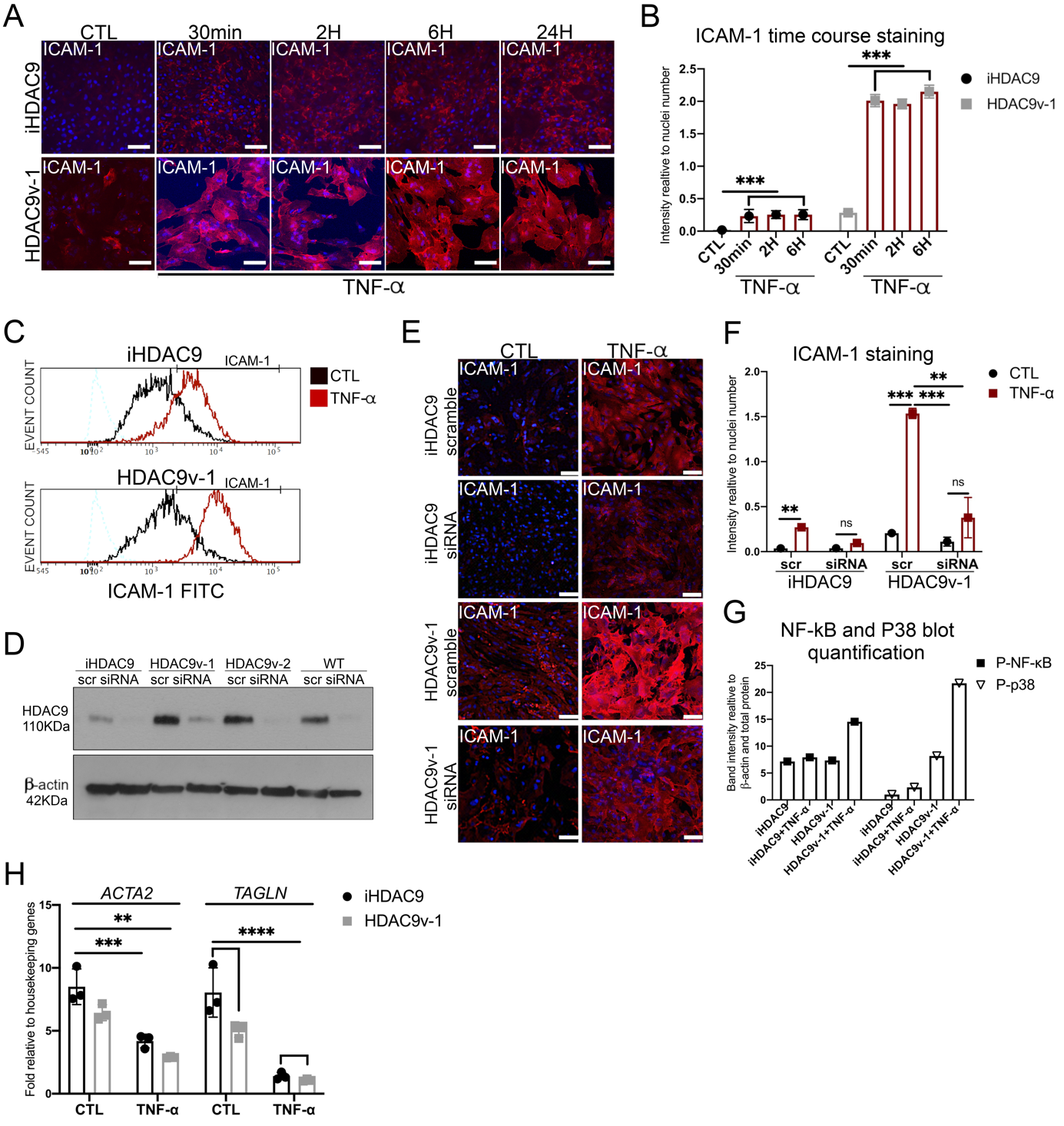


**Supplementary Figure 4. ICAM-1 response upon TNF-α stimulation in risk, controls and siRNA-mediated knock-down SMC. A)** Immunostaining for ICAM-1 in iHDAC9 and HDAC9v-1 SMC control (CTL) and upon stimulation with TNF-α after 30 minutes, 2, 6 and 24 hours. **B)** Quantification of ICAM-1 staining levels relative to cells number (nuclei). **C)** Flow cytometry analysis for ICAM-1 in control (CTL) and SMC upon TNF-α treatment for iHDAC9 (15.7% positive) and HDAC9v-1 line (34.8% positive). **D)** Cropped blot of HDAC9 protein in WT, iHDAC9, HDAC9v-1 and HDAC9v-2 SMC, upon transfection with scramble siRNA (scr) or specific siRNA for HDAC9 (siRNA). **E)** Immunostaining for ICAM-1 in iHDAC9 and HDAC9v-1 SMC either transfected with scramble or HDAC9 siRNA untreated and treated with TNF-α and **(F)** quantification of ICAM-1 staining relative to cells number (nuclei)**. G)** Quantification of phospho-levels of NF-κB and p38 in **Figure 5D**. **H)** RT-PCR for SMC markers, *ACTA2* and *TGLN* in iHDAC9 and HDAC9v-1 SMC control (CTL) and upon stimulation with TNF-α. Nuclei were stained with DAPI. Scale bar=100μm. The result is representative of three independent experiments (means ± SD). ***P* < 0.01; ****P* < 0.001. Statistical analysis was performed by 2-way ANOVA with Tukey’s multiple comparison test.

**Supplementary Figure 5. Full-length blots**


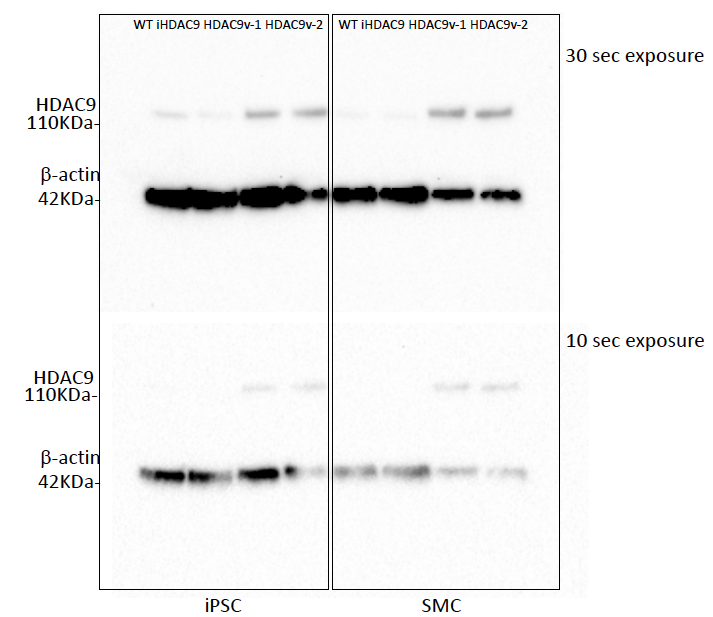


Full-length blot Figure 1E.


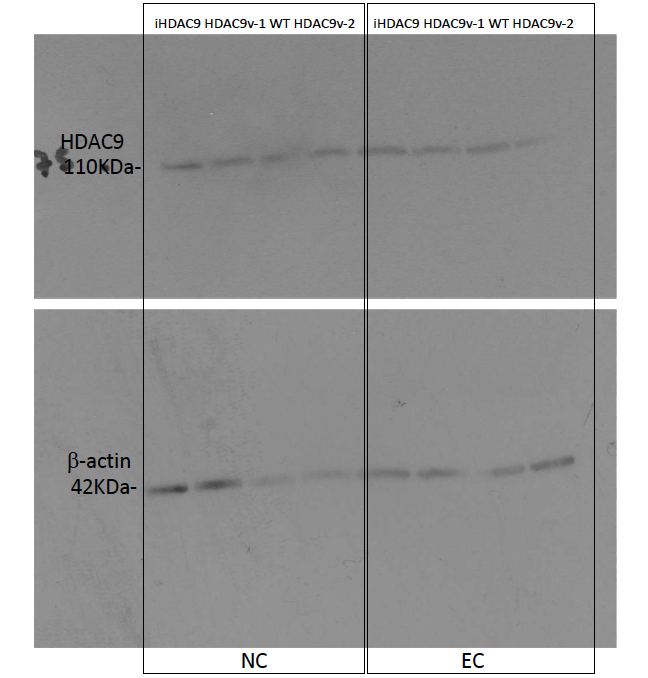


Full-length blot Supplementary Figure 2C.

**
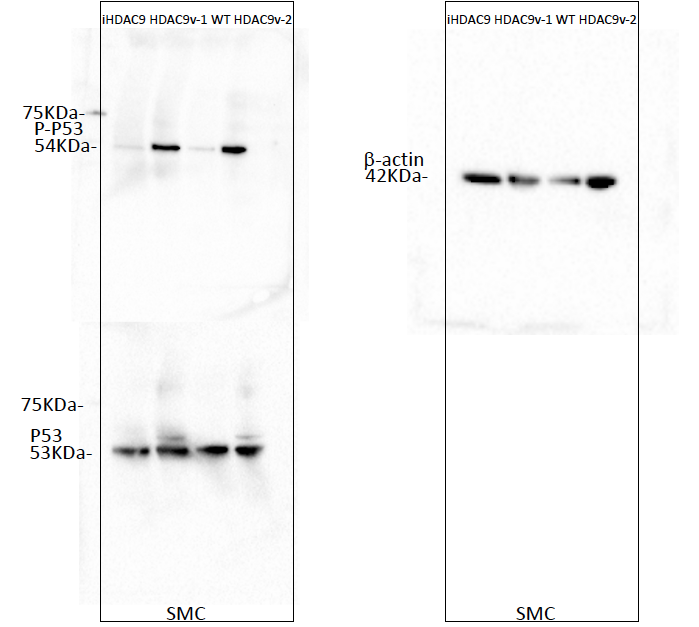
**

Full-length blot Figure 2D


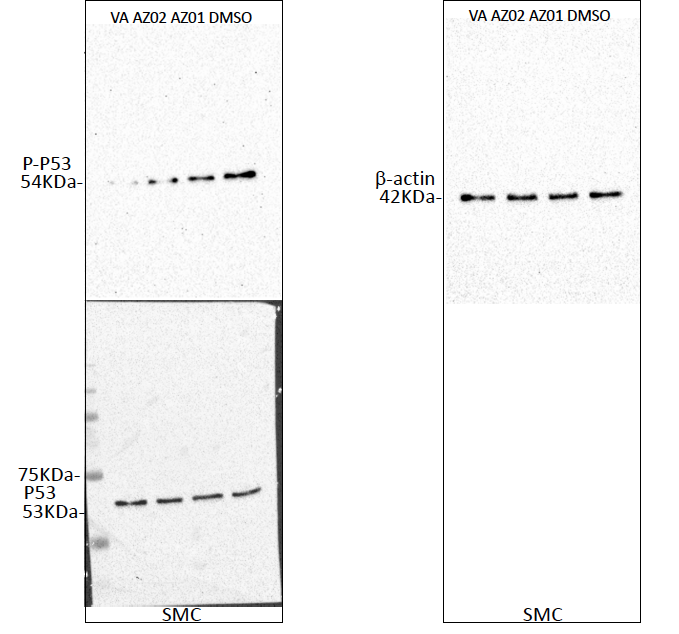


Full-length blot Figure 4E.


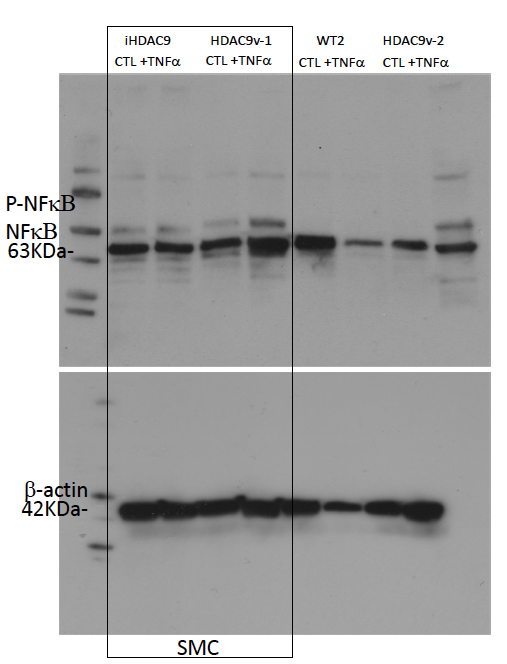

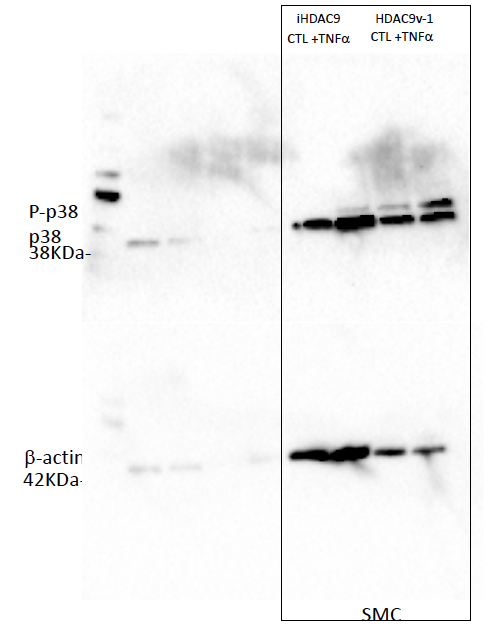


Full-length blot Figure 5D.


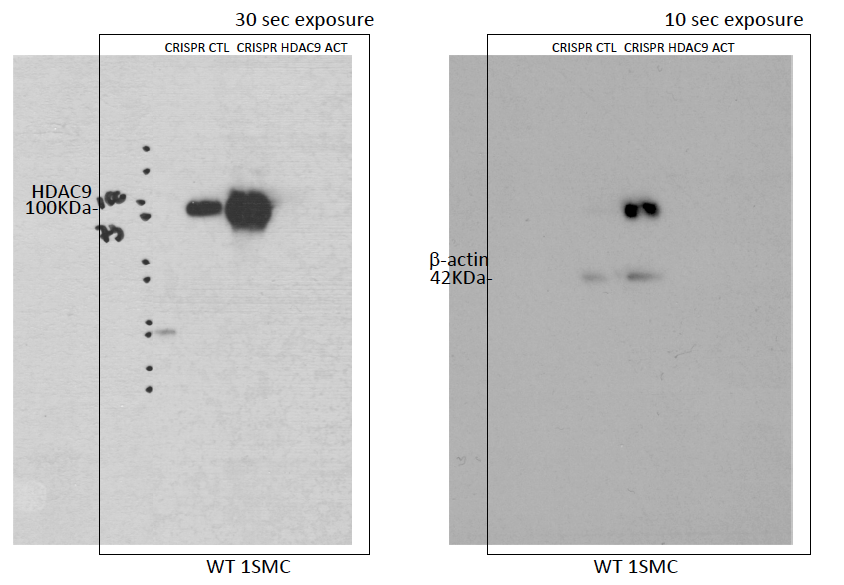


Full-length blot Figure 7A.

**
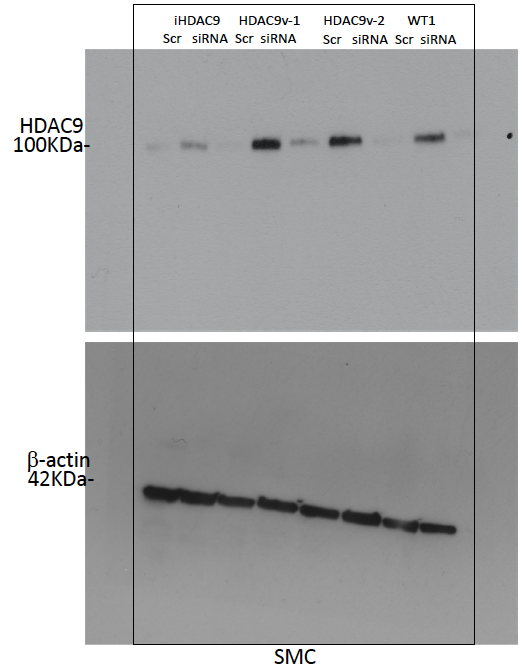
**

Full-length blot Supplementary Figure 4C.
